# Supplementary material for: Time-resolved spectroscopic and electrophysiological data reveal insights in the gating mechanism of anion channelrhodopsin
Source: Commun Biol. 2021 May 14;4:578. doi: 10.1038/s42003-021-02101-5 (PMC8121809; doi:10.1038/s42003-021-02101-5)
Supplement: Supplementary file 2 — Supplementary Information [file 42003_2021_2101_MOESM2_ESM.pdf]

Supplement for

Time-resolved spectroscopic and electrophysiological data reveal insights in the gating mechanism of anion channelrhodopsin

Authors

Max-Aylmer Dreier[1,2], Philipp Althoff[1,2], Mohamad Javad Norahan[1,2], Stefan Alexander Tennigkeit[1,2], Samir F. El-Mashtoly[1,2], Mathias Lübben[1,2], Carsten Kötting[1,2], Till Rudack[1,2,*], Klaus Gerwert[1,2,*]

Affiliations

[1] Biospectroscopy, Center for Protein Diagnostics (ProDi), Ruhr University Bochum, Bochum, Germany

[2] Department of Biophysics, Ruhr University Bochum, Bochum, Germany

*Corresponding authors:

Till Rudack; till.rudack@rub.de

Klaus Gerwert; klaus.gerwert@rub.de

Supplementary note 1: Kinetic analysis. For kinetic analysis the sample was allowed to fully return to its ground state for 60 s before being excited again. Data from time resolved FTIR and UV/VIS measurements was subjected to global fit analysis that yielded 6 transitions (T_1-T_6) with half-lives that match those previously published[1,2] allowing us to annotate the transitions with channel opening and closing (Figs. 2, S2, S3 and, Table 1). T_1 represents the decay of K with a half-live of 450 ns, which is in accordance with the transition of 3 µs observed in our UV/VIS measurements and 3.5 µs reported by others[2]. The next two processes (T_2 and T_3) are resolved to transitions within L that are observed in the infrared with 18 µs and 1.9 ms. Both have not been detected in previous spectroscopic measurements, but are in good agreement with formation of the conducting pore observed at 51 µs and 1.3 ms by electrophysiological measurements[1]. This concordance allows to assign our spectroscopic half-lives to the formation of the channel conducting state. In fact, in our UV/VIS measurements we also identified in total six transitions (Fig. S4) including two additional ones for the formation of the conducting state (23 µs and 2.3 ms). In the IR the two processes are dominated by alterations in the amide regions and in the carbonyl region. Retinal bands in the FTIR are hardly affected by these transitions and neither are the UV/VIS traces that also arise from retinal absorption. Instead the rise of a prominent band at 1558 cm^{-1} can be observed. Since this band does not arise concomitant with the C=C-vibrational band at 1529 cm^{-1} of the retinal it is likely to originate from amide II vibrations that are directly affected by opening and closing of the channel as this band nicely follows channel opening and closing. Also, the negative rise of the prominent band at 1644 cm^{-1} correlates with the opening of the channel. That, and its prominent position let us annotate this band to changes in the amide I band that likely represent major rearrangements of the backbone of the protein. The following two processes (T_4 and T_5) represent the formation and decay of M with 35 ms and 107 ms, which is also in agreement with electrophysiological measurements, and is associated with the decay of the conducting state (33 ms and 165 ms)[1]. This can nicely be observed in the UV/VIS kinetics in Fig. S4. In addition, the half-lives suit with the published UV/VIS measurements (15 ms and 87 ms)[2]. The corresponding global fit-derived FTIR amplitude spectra for the four aforementioned half-lives T_2 to T_5, which correspond to formation and decay of the conducting state, can be seen in Fig. S2, which allow to derive state-specific marker bands. Rise of M sees the decay of the 1184 cm^{-1} band and also of a band at 1303 cm^{-1} that might well arise from the N-H in-plane bending mode of the SB in L as in bR[3]. The final process (T_6) represents the decay of an N/O mixed species back to the ground state with 4.4 s, which also fits with the 2.6 s from published UV/VIS measurements[2].

UV/VIS data of *Gt*ACR1 (Fig. S4) nicely complement the FTIR data as we again find 6 transitions with similar half-lives. A major K → L transition is observed with 3 µs. The two half-lives associated with channel opening resemble this first transition but are much smaller in amplitude, underlining that channel opening is a process taking place within the L-intermediate. M-formation occurs concomitant with depletion of K and L due to the equilibrium between them. M decays to a broad band around 520 nm that very much resembles the ground state and has a shoulder at 450 nm that can be assigned to a N-state. N/O decays back to the ground state with a half-life of 3.5 s, however the amplitude of this transition is small. Our UV/VIS data go beyond those already published [2] as they provide the transitions necessary to describe channel gating as well.

In the carbonyl region of the FTIR spectra, a band at 1716 cm^{-1} appears as well as negative bands at 1708 cm^{-1} and 1725 cm^{-1}. These bands are likely to originate from glutamates or aspartates changing protonation or hydrogen-bonding states. The 1708 cm^{-1} band was assigned to Glu-68 at 170 K earlier[4] and is visible already in the sub-microsecond time range. The kinetic of this band shows a stepwise decay of the band until it vanishes upon channel closing. The band could simply arise from a protonated, hydrogen bonded Glu-68 that experiences changes in hydrogen bonding strength. The putative corresponding difference band at 1716 cm^{-1} however does not exactly counterbalance the band

at 1708 cm^{-1}. The reason for this might be a continuum band that seems to be present in the spectra prior to channel opening. Continuum bands arise from hydrogen-bonded networks of amino acids and water molecules that show very broad absorption bands in the IR[5]. The difference spectra in Fig. S6 show a broad negative absorption between 1700 cm^{-1} and 1800 cm^{-1} that resembles the continuum band of bacteriorhodopsin and seems to decay before and upon the opening of the cannel. This can also be observed in the amplitude spectrum of the 18 µs half-life in Fig. S2. Nonetheless, an early deprotonation of Glu-68 and reprotonation upon channel opening cannot be excluded. Mechanistically it would make sense to neutralize a negative charge to open an anion channel. Two bands at 1740(-)/1732(+) cm^{-1} were assigned to Asp-234 at 77 K in another study[6] that do not clearly correspond to bands in our measurements at ambient temperature. The difference spectrum at 200-400µs (Fig. S6) shows bands that might correspond to those bands assigned to Asp-234 which would indicate a change in hydrogen bonding upon formation of the conducting state. The amplitude spectra in Fig. S2 however do not clearly show the formation of these bands. Band assignments at ambient temperature and with time-resolved data is required to clarify the roles of Glu-68 and Asp-234.

Supplementary note 2: Isotopic labelling. In Fig. S16 FTIR difference spectra of unlabeled and $^{13}C_{14}$-$^{13}C_{15}$ retinal-labeled *Gt*ACR1 and *Cr*ChR2 are compared. Since neither the C=N-*syn* band at 1154 cm^{-1} nor any other band in this region exhibiting the characteristic shifts upon isotopic labelling is found, FTIR argues strongly against the existence of a C=N-*syn* species in the excited state of *Gt*ACR1. Raman spectra of the dark state and the excited photostationary state (P_{stat}) are shown in Fig. S17. Unlabeled P_{stat} shows no band in the region of 1154 cm^{-1} that would indicate a C=N-*syn* species in the photoproduct. Also, the P_{stat} spectra of $^{13}C_{14}$-$^{13}C_{15}$ retinal-labeled sample show no band with the characteristic ~14 cm^{-1} downshift. It should be noted however, that the band at 1154 cm^{-1} is almost invisible in P_{stat} spectra of *Cr*ChR2 and only becomes visible upon $^{13}C_{14}$-$^{13}C_{15}$ labeling of the retinal.

$^{13}C_{14}$-$^{13}C_{15}$ labeling of the retinal causes significant shifts. The negative band at 1234 cm^{-1} is slightly downshifted upon $^{13}C_{14}$-$^{13}C_{15}$ labeling resembling the band at 1254 cm^{-1} of the light-adapted ground state of bR[7] and the band at 1234 cm^{-1} of *Cr*ChR2[8]. In bR and *Cr*ChR2 this band has been assigned to the anti-symmetric combination of the C_{12}-C_{13} + C_{14}-C_{15}-stretching and the H_{14} rocking vibration and is therefore assigned to the same combination mode here for *Gt*ACR1. The band at 1201 cm^{-1} has been assigned to the C_{14}-C_{15} stretch vibration of the all-*trans*,C=N-*anti* retinal[9] due to its analogous position in bR[7]. This assignment can be confirmed due to the 27 cm^{-1} downshift upon $^{13}C_{14}$-$^{13}C_{15}$ labeling that is visible in the FTIR difference spectra in Fig. S16 as well as in the Raman spectra in Fig. S17A. The band at 1161 cm^{-1} in Fig. S16B was assigned to a localized C_{10}–C_{11} stretching mode in analogy to the 1168 cm^{-1} band of bR[9]. The band seems to vanish in our FTIR difference spectra upon $^{13}C_{14}$-$^{13}C_{15}$ labeling. The same has been observed in Raman spectra of *Cr*ChR2[8] and of crystals of all-*trans* retinal with a protonated Schiff base[10]. The Raman spectrum in Fig. S17A however shows a prominent band at 1158 cm^{-1} that downshifts to 1154 cm^{-1} upon $^{13}C_{14}$-$^{13}C_{15}$ labeling. This agrees well with the 1168 cm^{-1} band of bR that downshifts by 3 cm^{-1} upon $^{13}C_{14}$-$^{13}C_{15}$ labeling[7]. The band at 1184 cm^{-1} is arises from the C_{14}-C_{15} stretching vibration of 13-*cis* retinal and was originally assigned in bR[11]. This band shifts by a few wavenumbers but is heavily overlaid by the shifted 1201 cm^{-1} band in FTIR spectra (Fig. S16B). The shift can be well observed in the Raman spectra of the illuminated sample (Fig. S17B).

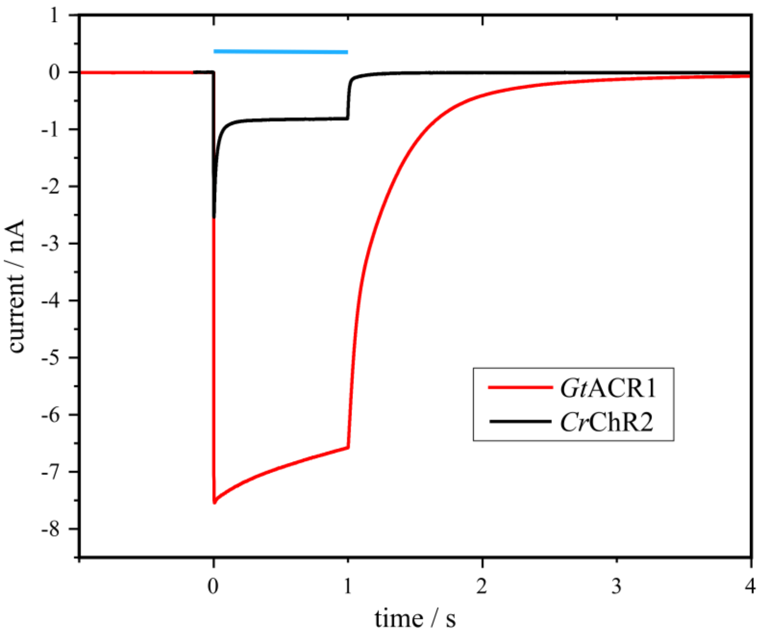

Supplementary Figure 1: Typical photocurrents as a response to a 1 s light pulse (blue bar) of *Cr*ChR2 and *Gt*ACR1 (data replotted from Govorunova et al., 2015, Fig. 1D[12]). *Cr*ChR2 shows a characteristic decay of the photocurrent upon continuous illumination. *Gt*ACR1 produces a significantly larger photocurrent compared to *Cr*ChR2 and does not display the described inactivation of the photocurrent. However, channel closing is much slower in *Gt*ACR1 compared to *Cr*ChR2.

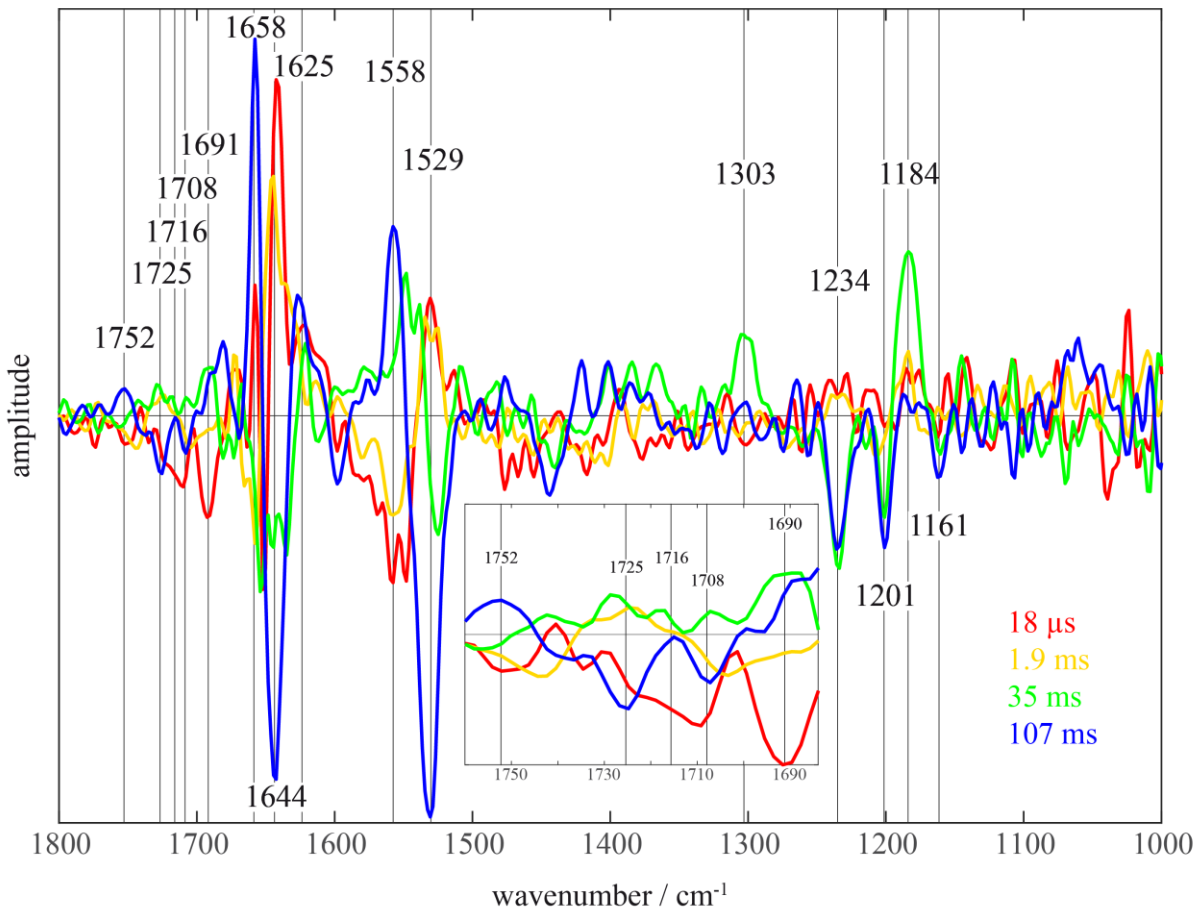

Supplementary Figure 2: FTIR amplitude spectra of channel opening and closing. The inset shows an enlargement of the carbonyl region. Both half-lives that account for channel opening (18 µs and 1.9 ms) show a high similarity. Channel opening is accompanied by the rise of a band at 1691 cm^{-1} and the negative rise of the band at 1644 cm^{-1} that is indicative of backbone movements[13]. In the carbonyl region bands at 1716 cm^{-1} and 1750 cm^{-1} rise, mostly with contributions of the 18 µs half-life. Fast channel closing (35 ms) is associated with decay of a band at 1303 cm^{-1} and the deprotonation of the SB visible at 1184 cm^{-1} due to the concomitant rise of M. Slow channel closing and decay of M are observed in the decay of the bands at 1644 cm^{-1} and 1691 cm^{-1}. Also, the bands of the retinal ground state at 1529 cm^{-1}, 1234 cm^{-1}, 1201 cm^{-1} and 1161 cm^{-1} decay with this half-life indicating the return of the retinal to its all-*trans* conformation. In the carbonyl region the 107 ms transition is associated with the decay of basically all visible bands. The 107 ms transition, and also the 35 ms transition, accounts for major reoccurrences of retinal ground state bands indicating reisomerization of the retinal to the all-*trans*,C=N-*anti* conformation in O. The reisomerization is also observed in two rates due to the equilibrium between L_2 and M, that causes the two-step process of channel closing observed in electrophysiological experiments. This reisomerization is also observed in UV/VIS measurements (Fig. S4).

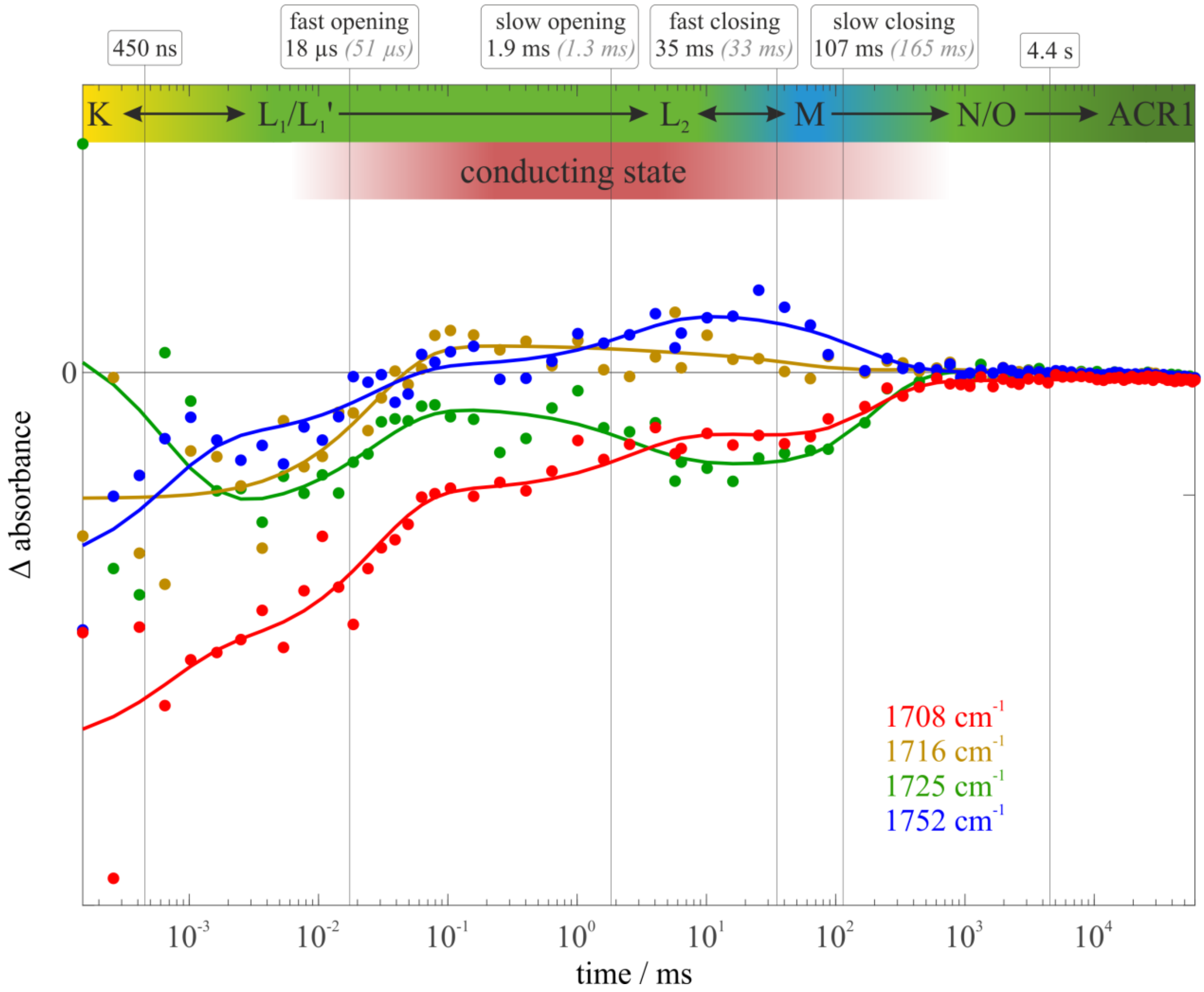

Supplementary Figure 3: FTIR kinetics of difference bands found in the carbonyl region. The assignment of the conducting state is based on the well according half-lives of the spectroscopic global fit analysis (bold black numbers and vertical solid black lines) and the published electrophysiologically determined half-lives of channel opening and closing[1] (gray italic numbers). The band at 1708 cm^{-1} is present at sub-microsecond times and decays stepwise. The decays leading to channel opening might represent a reprotonation of the residue. However, multiple bands seem to be affected by what might be a continuum band, that decays with 450 ns and partly with 18 µs (see also Fig S6). The bands then show some alterations in intensity before decaying with the slow channel closing.

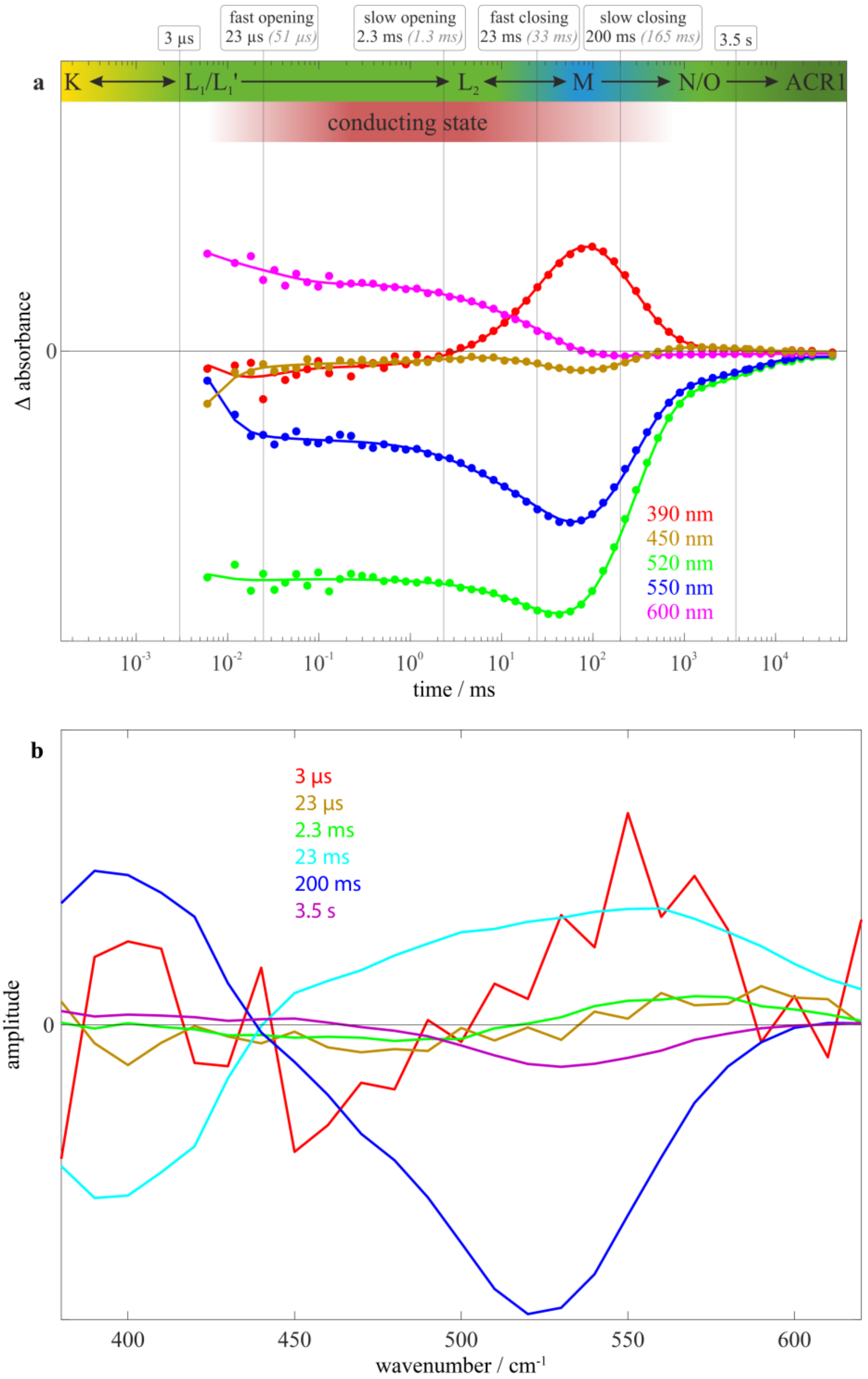

Supplementary Figure 4: UV/VIS analysis of the *Gt*ACR1 photocycle. The first observed transition is from K to L_1 that can be seen by the decay of a ~550 nm species to a ~450 nm species. The 23 µs and 2.3 ms transitions have very small amplitudes and basically reflect minor contributions of the same sort as the 3 µs process due to the equilibria between the intermediates. The 23 ms half-life reflects the rise of the M intermediate at ~390 nm. The decaying band in **b** for this transition is very broad, again due to the equilibria. M then decays to N/O with 200 ms. Due to the assumed reisomerization the absorption of this intermediate is low. The resulting amplitude spectrum in **b** shows an apparent decay to the ground state at ~520 nm with a shoulder at ~450 nm. This can also be observed in **a** in the 450 nm kinetic. The last half-life of ~3,5 s reflects the decay of this N/O intermediate with a low amplitude in the UV/VIS due to the retinal conformation. The assignment of the conducting state is based on the well according half-lives of the spectroscopic global fit analysis (bold black numbers and vertical solid black lines) and the published electrophysiologically determined half-lives of channel opening and closing[1] (gray italic numbers).

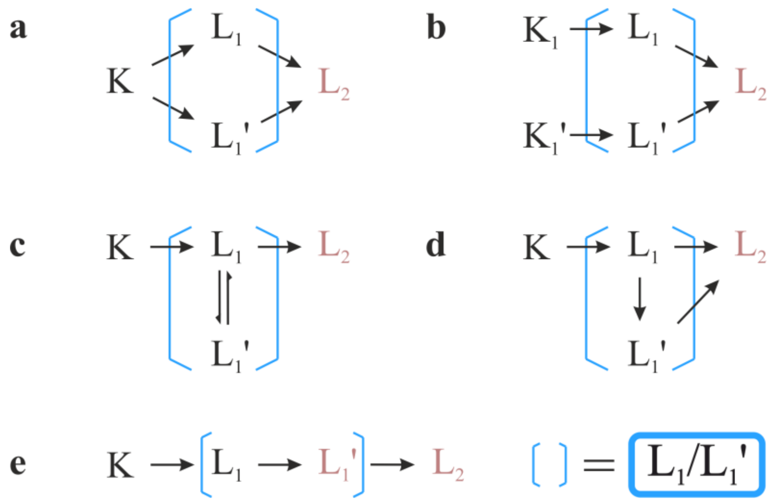

Supplementary Figure 5: Possible kinetic models describing the relations between the K, L_1 and L_2 intermediates. Open states are indicated by red font color. In **a** and **b** L_1 and L_1’ are two independent intermediates that both decay to L_2 with different half-lives. In **c** L_1 and L_1’ are in a slow equilibrium that decays to L_2 with two different half-lives. In **d** L_1 decays to L_2 or to L_1’ and L_1’ in turn decays to L_2 resulting in 2 half-lives for the L_2 formation. **e** is a simple succession of the observed transitions where L_1 decays to an open state (L_1’). Alterations of this open state then form the L_2 state. The five different types of L1 and L1’ indicated by blue parenthesis are simplified denoted as L1/L1’ in Fig. 4.

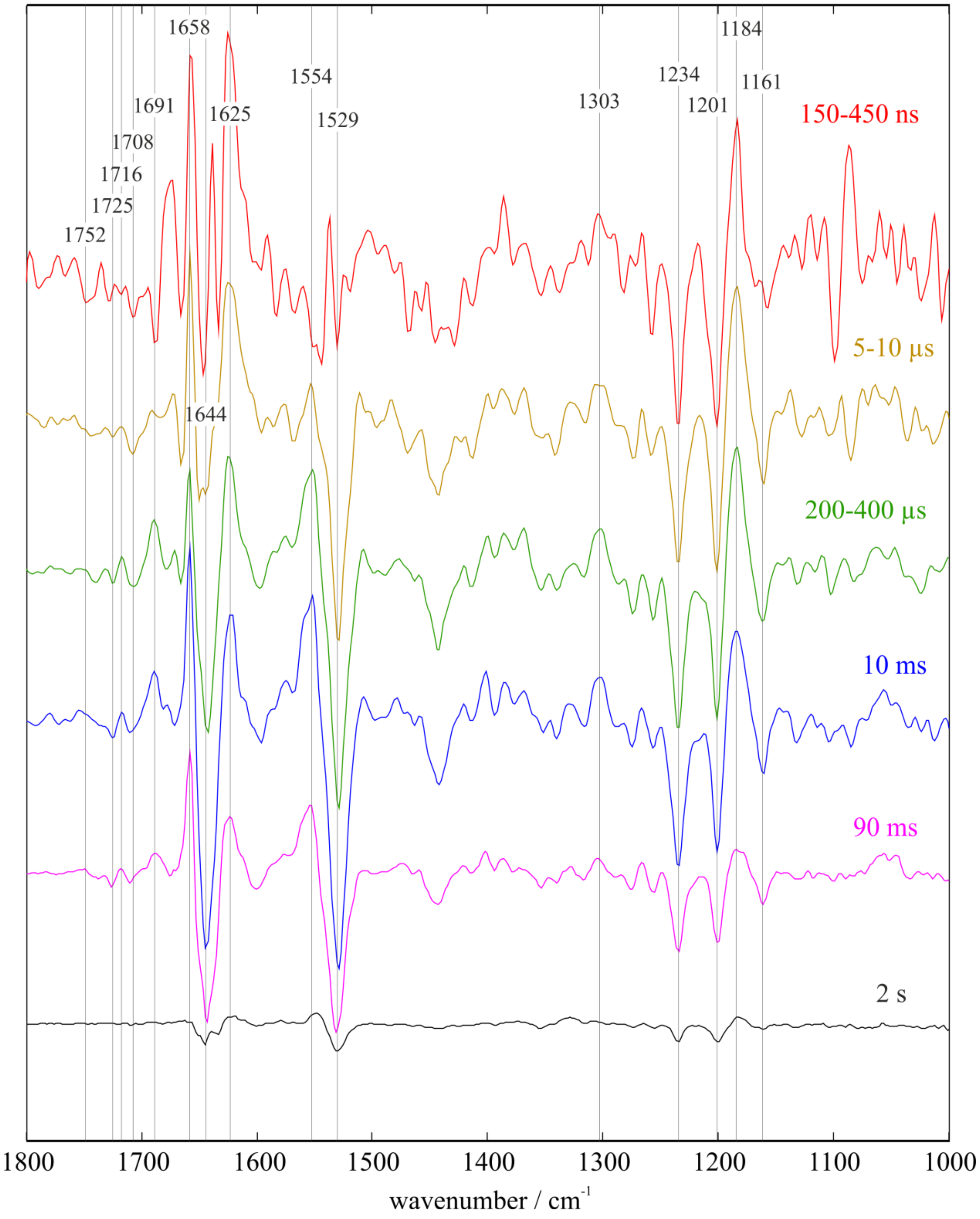

Supplementary Figure 6: FTIR intermediate-minus-ground state difference spectra of *Gt*ACR1 at different time points after excitation.

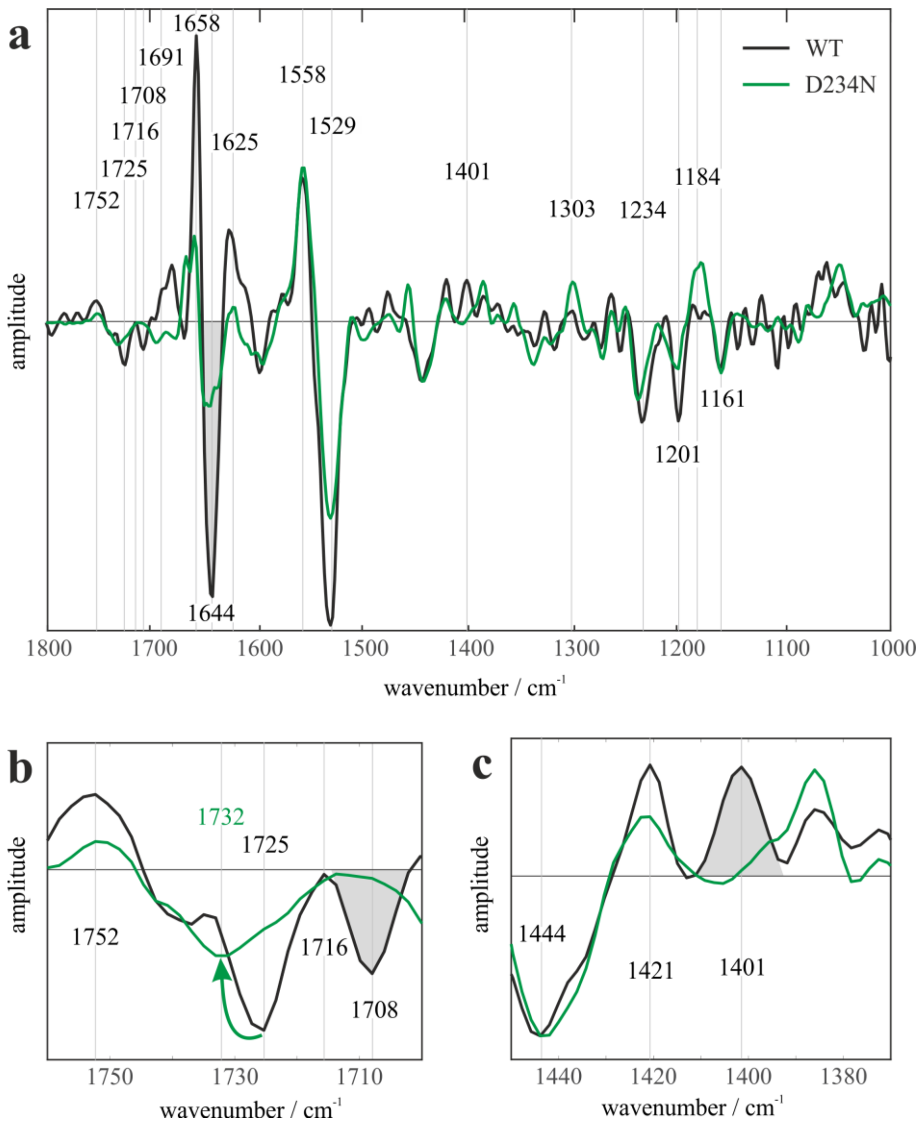

Supplementary Figure 7. WT and D234N amplitude difference spectra of slow channel closing (M-decay): Comparison of amplitude spectra of WT and D234N with half-lives of 107 ms and 1.3 s, respectively. **a** The highlighted band at 1644 cm^{-1} that was assigned to channel opening is significantly lower in D234N compared to WT. This lower intensity supports the band assignment as the D234N has significantly lower conductivity than WT as shown by electrophysiological measurements[2]. **b** Enlargement of the carbonyl region. The band at 1725 cm^{-1} shifts in D234N to 1732 cm^{-1} and loses intensity. As the band is still present in D234N the band is not evoked by Asp-234. The absence of the band at 1708 cm^{-1} which was assigned to Glu-68 in D234N suggests that there is no protonation change of Glu-68 in D234N. **c** Enlargement of the region that was identified by theoretical IR spectroscopy to contain possible bands of deprotonated Asp-234. The highlighted band at 1401 cm^{-1} is not present in D234N and is therefore a possible candidate to reflect deprotonated Asp-234. The data is scaled according to the amplitude of the M decay at wavenumber 1444 cm^{-1}.

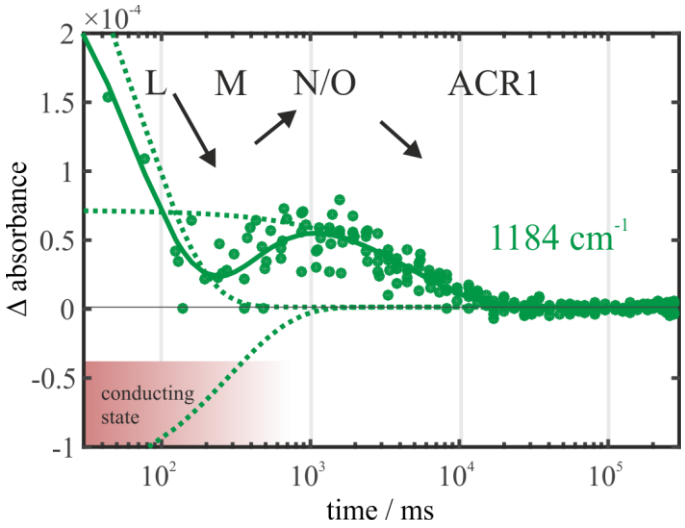

Supplementary Figure 8: FTIR kinetics of 1184 cm^{-1} band in detergent solubilized sample. Rise and decay of the N/O intermediate can be observed under these conditions.

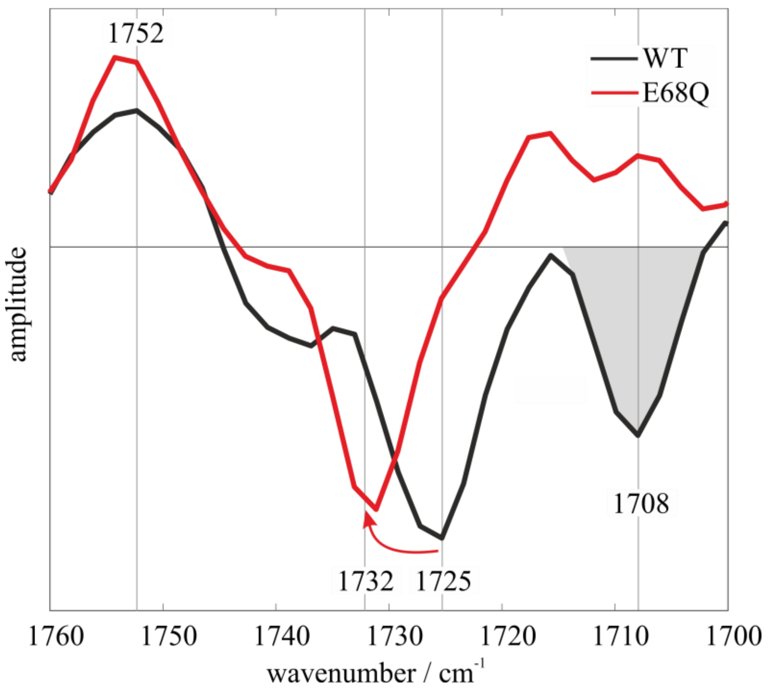

Supplementary Figure 9. Carbonyl region of WT and E68Q amplitude difference spectra of slow channel closing (M-decay): Comparison of amplitude spectra of WT and E68Q with half-lives 107 ms and 46 ms, respectively. The negative band at 1708 cm^{-1} is absent in E68Q, confirming its band assignment to protonated Glu-68. In addition, the band at 1725 cm^{-1} shifts to 1732 cm^{-1} reflecting an impact of Glu-68 on the band but it cannot be evoked by the carbonyl group of Glu-68. The data is scaled according to the amplitude of the M decay at wavenumber 1444 cm^{-1}.

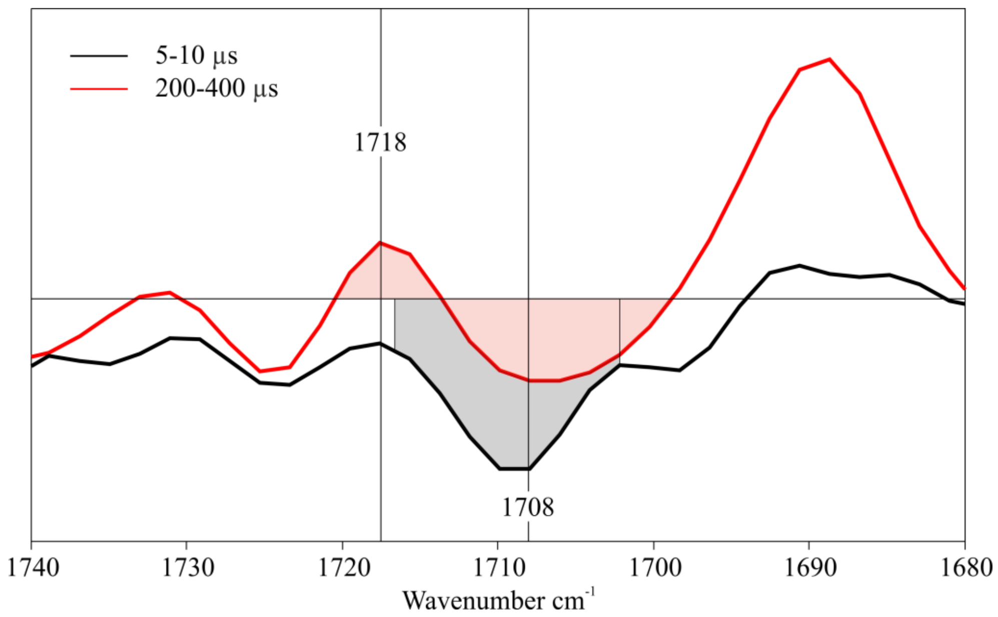

Supplementary Figure 10: Carbonyl region of FTIR difference spectra of *Gt*ACR1 at 5-10 µs (black) and at 200-400 µs (red) after excitation. The negative difference band at 1708 cm^{-1} visible in the 5-10 µs spectrum indicates deprotonated Glu-68. The band partially decays and concomitant a positive difference band at 1718 cm^{-1} appears as visible in the 200-400 µs spectrum. This indicates the transition to a reprotonated state that differs from the ground state in the hydrogen bonding pattern of Glu-68.

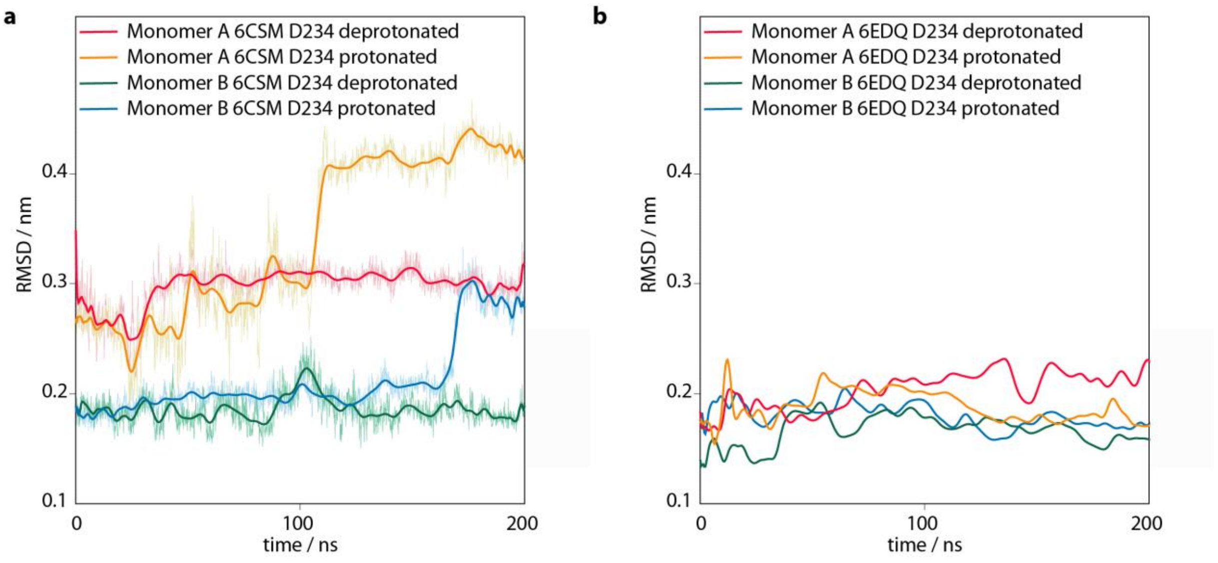

Supplementary Figure 11. Root mean square deviation (RMSD) analyses. The backbone RMSDs for each of the monomers of the trajectories of the 200 ns production runs initiated by the X-ray structure with PDB-ID 6CSM[6] (**A**) and 6EDQ[14] (**B**). The reference structure for RMSD calculations is the respective optimized starting structure before heating of the system. The plots are smoothened using Bezier-curves. For clarity, unsmoothed data points are only shown in (A). The simulation initiated by 6EDQ reflect stable and equilibrated RMSD curves whereas the ones based on 6CSM are still fluctuating. Therefore, further analysis was only performed for simulations based on 6EDQ.

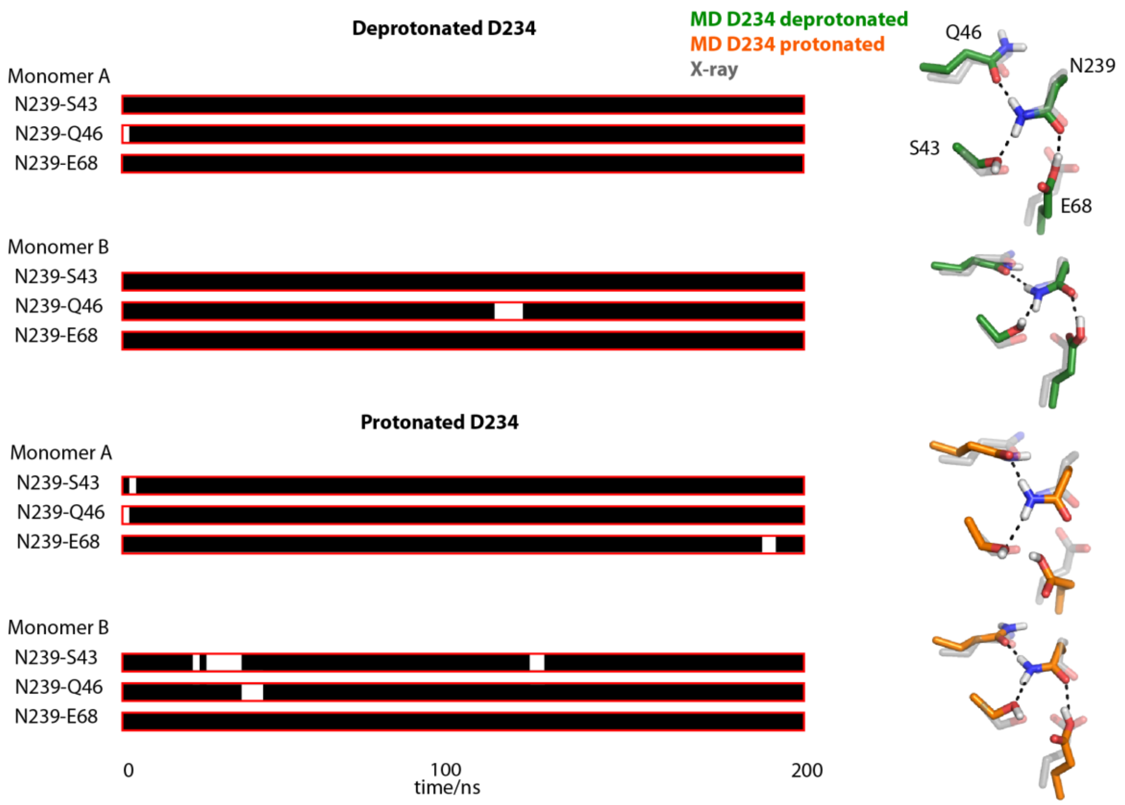

Supplementary Figure 12. Central constriction site hydrogen bond interaction network. The left panel shows the contact pattern of the central constriction side within the course of simulation initiated by 6EDQ. A black bar reflects a hydrogen bond and a white bar no bond. The right panel shows a zoom on the central constriction site structural models. The representative structure of the whole simulation system (green Asp-234 deprotonated and orange Asp-234 protonated) is compared with the X-ray structure. The central constriction site hydrogen bond network is stable independent of the protonation state of Asp-234 and reflects the network indicated by the X-ray structures 6EDQ and 6CSM.

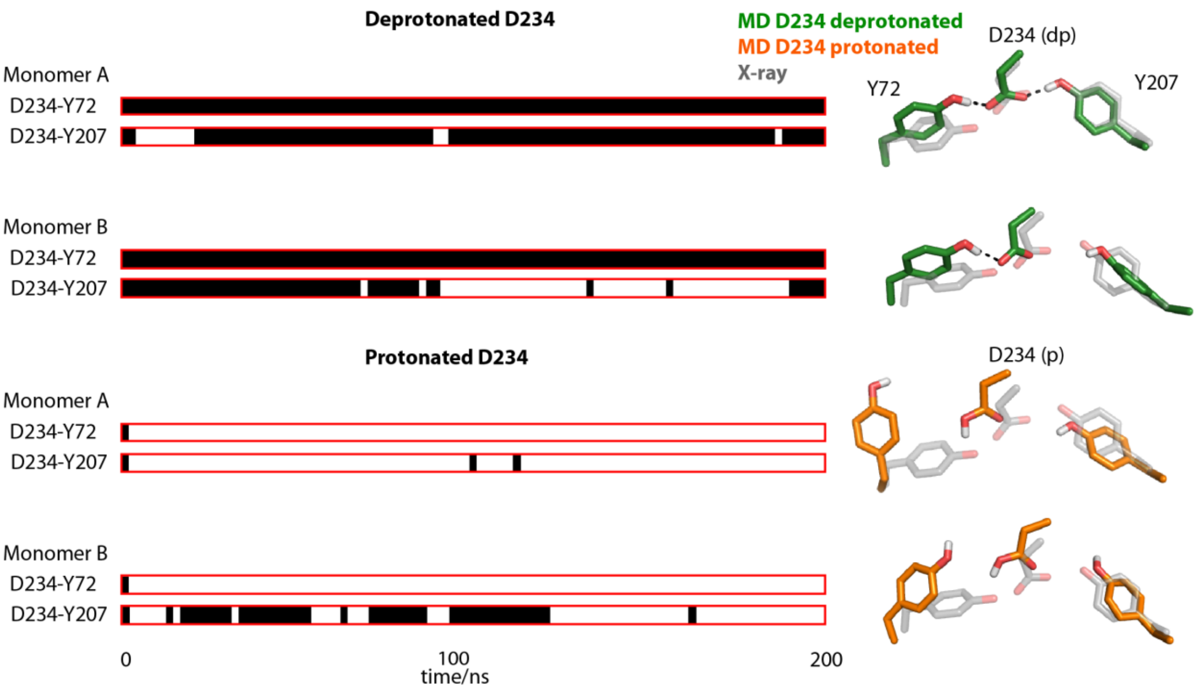

Supplementary Figure 13. Asp-234 hydrogen bond interaction network. The left panel shows the contact pattern between Asp-234, Tyr-72, and Tyr-207 within the course of simulation initiated by 6EDQ. A black bar reflects a hydrogen bond and a white bar no bond. The right panel shows a zoom on the involved residues in the structural models. The representative structure of the whole simulation system (green Asp-234 deprotonated and orange Asp-234 protonated) is compared with the X-ray structure. The hydrogen bond network is stable for deprotonated Asp-234 and reflects the network indicated by the X-ray structures 6EDQ and 6CSM. In the simulations with protonated Asp-234 the hydrogen bond network between the three residues abolishes and the converged structure does not reflect the position of Tyr-72 within the available X-ray structures.

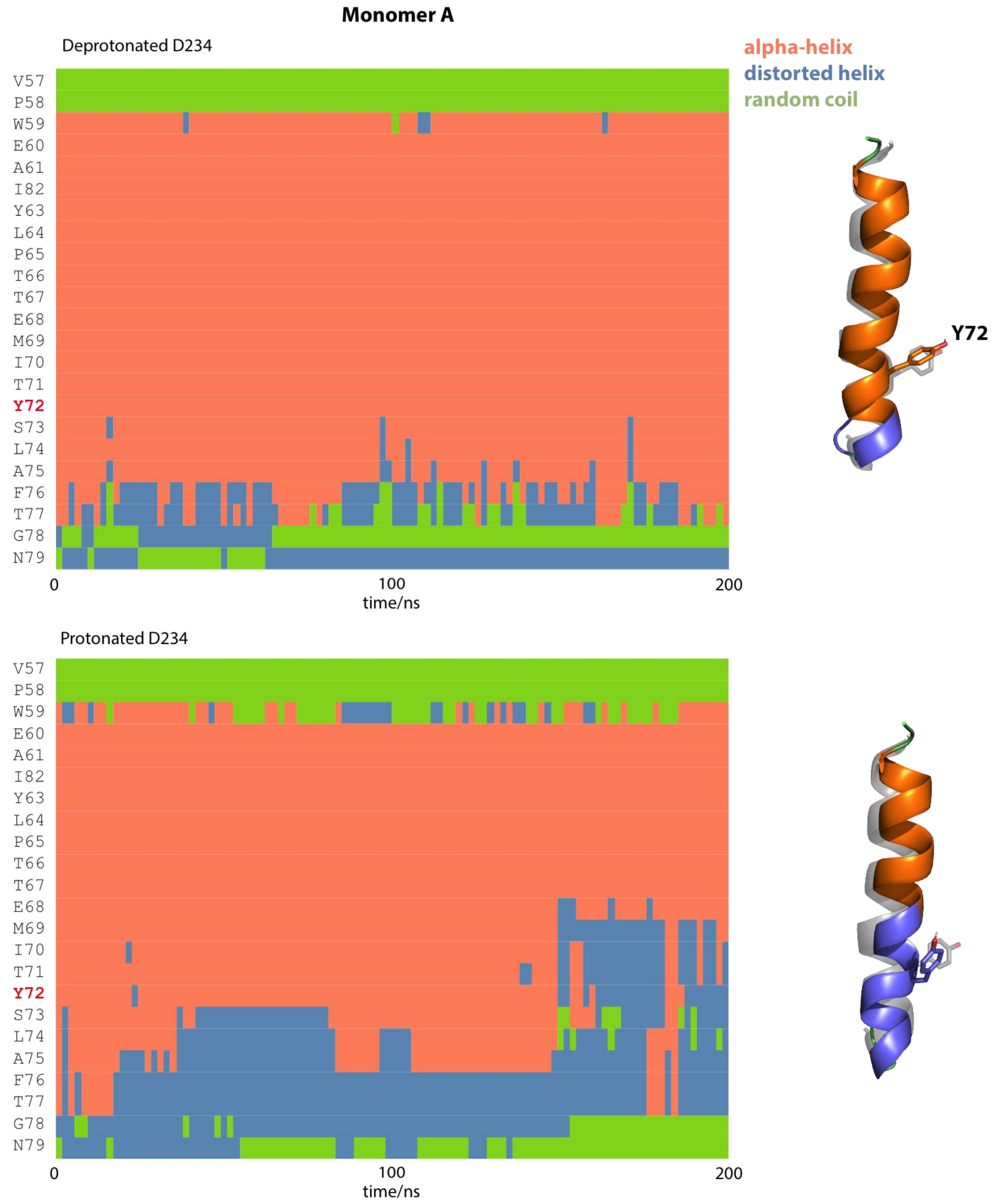

Supplementary Figure 14. Secondary structure analyses of monomer A. Results of simulations trajectories for monomer A based on 6EDQ for protonated and deprotonated Asp-234 are compared. The left panel gives the result of a DSSP secondary structure analysis for helix 2 over the course of simulation time. The right panel shows a zoom on helix 2 of a representative structure of the simulation trajectory (colored by secondary structure elements) compared with the X-ray structure (gray). For deprotonated Asp-234 only the upper part of helix 2 get distorted and the region around Tyr-72 stays a stable helix, whereas for protonated Asp-234 also the region around Tyr-72 gets distorted.

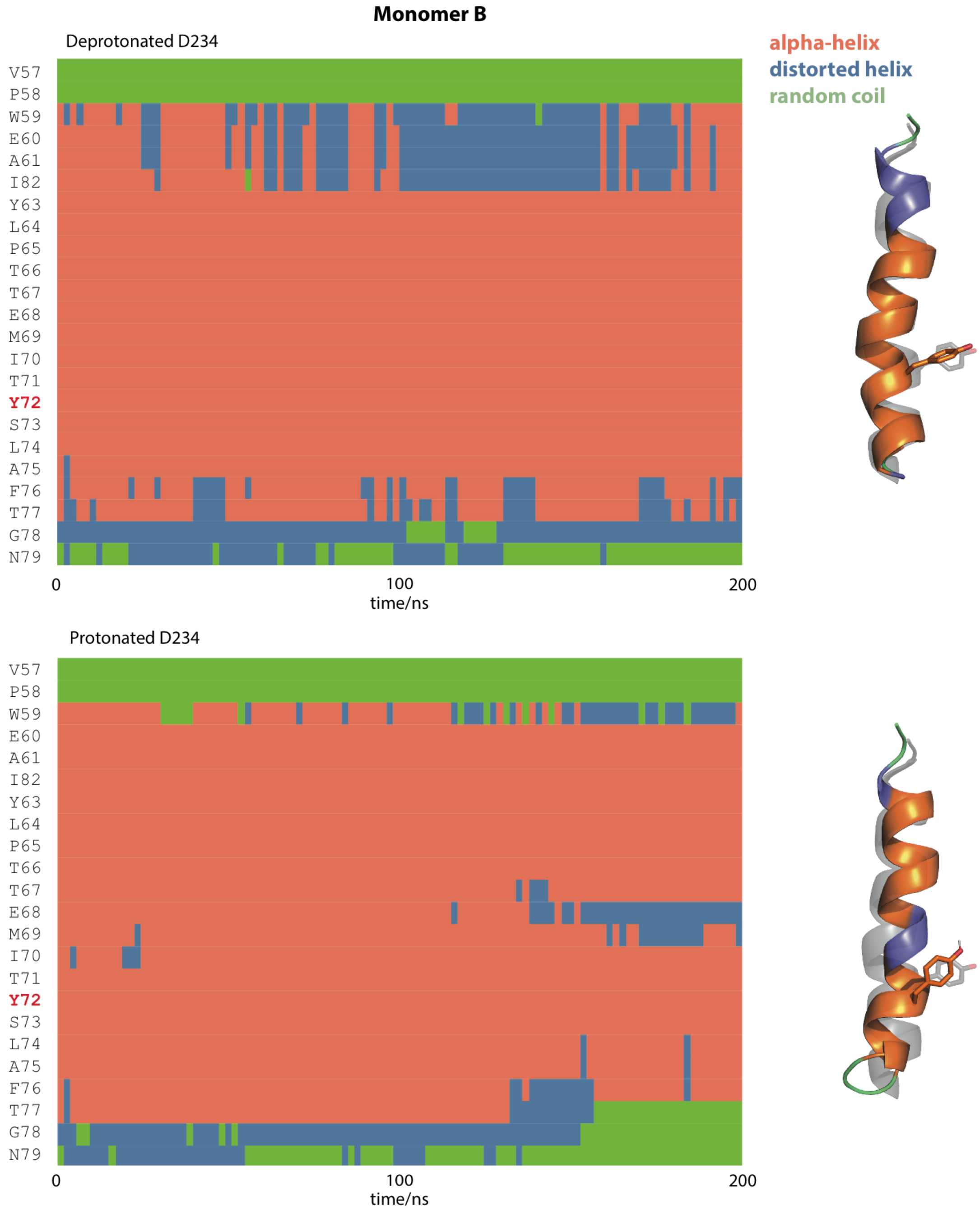

Supplementary Figure 15. Secondary structure analyses of monomer B. Results of simulations trajectories for monomer B based on 6EDQ for protonated and deprotonated Asp-234 are compared. The left panel gives the result of a DSSP secondary structure analysis for helix 2 over the course of simulation time. The right panel shows a zoom on helix 2 of a representative structure of the simulation trajectory (colored by secondary structure elements) compared with the X-ray structure (gray). For deprotonated Asp-234 the lower part of helix 2 fluctuates between a normal and a distorted helix. The region around Tyr-72 stays a stable helix. For protonated Asp-234 the distortion effect in the region around Tyr-72 is less drastic than observed for monomer A (Fig. S14) but a distortion in the middle of the helix below Tyr-72 is observed leading to a destabilization of the whole helix.

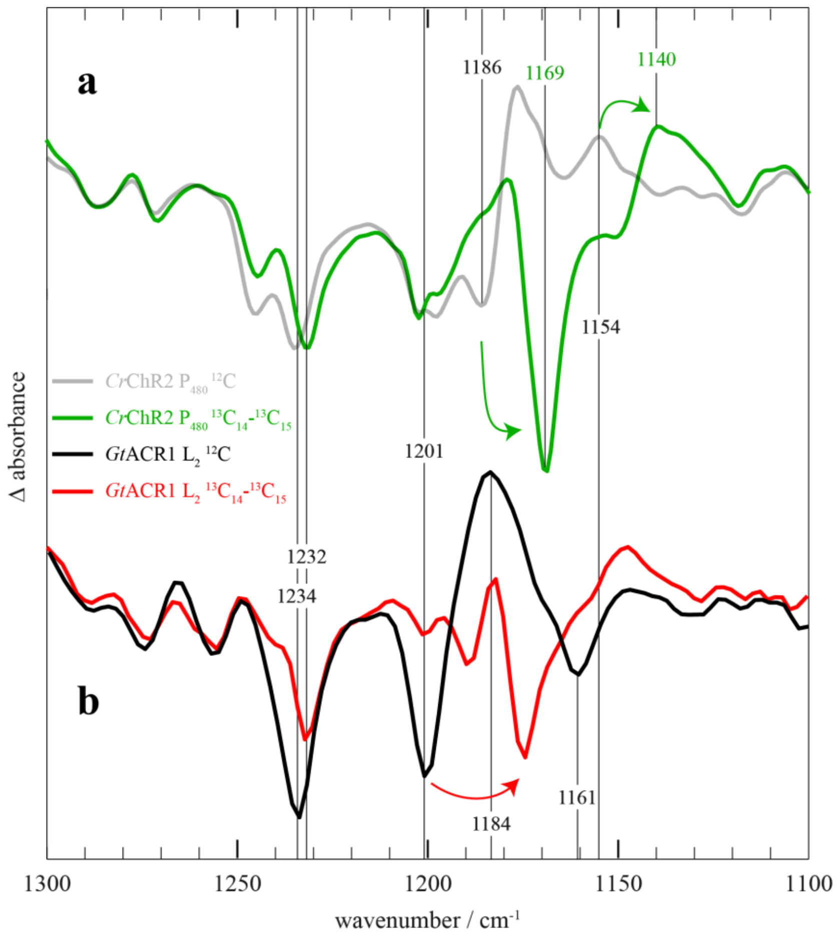

Supplementary Figure 16: a Comparison of a P_{480} minus D_{470} difference spectrum of *Cr*ChR2 with unlabeled and $^{13}C_{14}$-$^{13}C_{15}$ labeled retinal. **b** Comparison of a L_2 minus ground state difference spectrum of *Gt*ACR1 with unlabeled and $^{13}C_{14}$-$^{13}C_{15}$ labeled retinal. The respective shifts of the C=N-*syn* band of *Cr*ChR2 at 1154 cm^{-1} are indicated. No C_{14}-C_{15} stretching mode is found in the fingerprint region of the difference spectra.

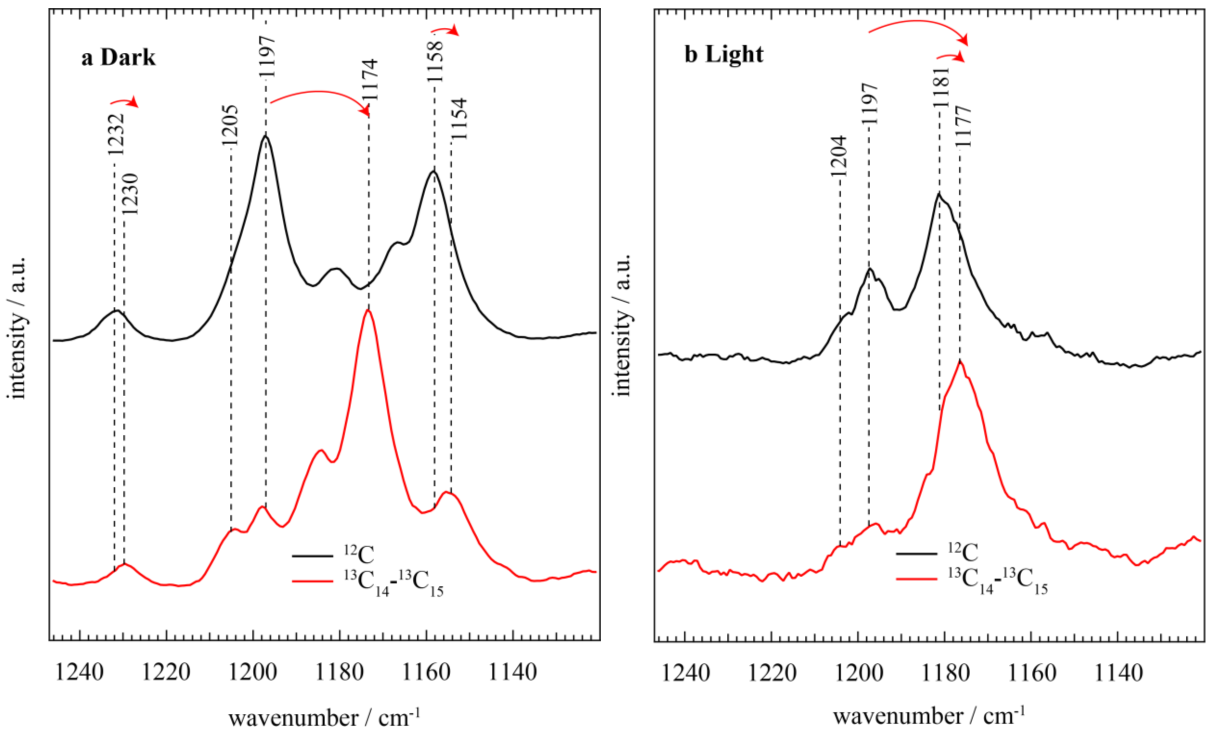

Supplementary Figure 17: a Raman spectra of *Gt*ACR1 in the dark. **b** Raman spectra of the illuminated state of *Gt*ACR1. Spectra were obtained by measuring under continuous illumination of the sample and subsequent scaling and subtraction of the dark state in (A).

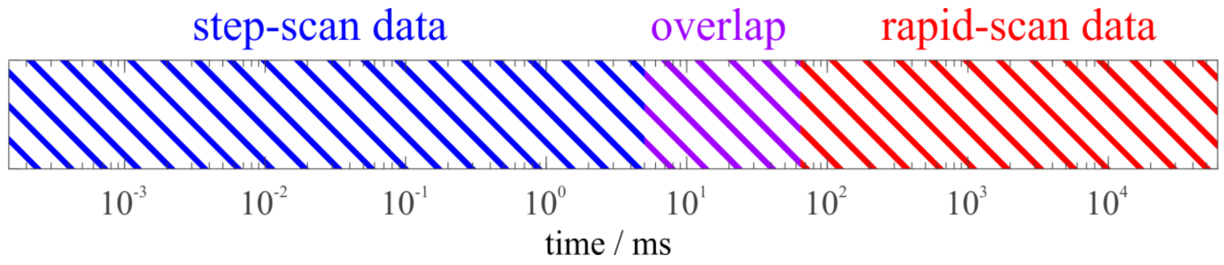

Supplementary Figure 18: Principle of combination of different FTIR data sets. Since step-scan measurements cannot be conducted over such long periods of time due to instabilities of the interferometer mirror the step-scan data were combined with rapid-scan measurements. These have a temporal resolution in the ms-timescale but can be conducted over long periods. To avoid discontinuities, we allowed for ~1 order of magnitude for overlap between both data sets. To ensure comparability of both data sets the sample was allowed to fully relax to its ground state in both measurements, resulting in equal laser flash repetition rates.

References

1. Sineshchekov, O. A., Govorunova, E. G., Li, H. & Spudich, J. L. Gating mechanisms of a natural anion channelrhodopsin. *Proc. Natl. Acad. Sci. U.S.A.* **112**, 14236–14241 (2015).
2. Sineshchekov, O. A., Li, H., Govorunova, E. G. & Spudich, J. L. Photochemical reaction cycle transitions during anion channelrhodopsin gating. *Proc. Natl. Acad. Sci. U.S.A.* **113**, E1993-2000 (2016).
3. Maeda, A., Sasaki, J., Pfefferlé, J.-M., Shichida, Y. & Yoshizawa, T. FOURIER TRANSFORM INFRARED SPECTRAL STUDIES ON THE SCHIFF BASE MODE OF ALL-trans BACTERIORHODOPSIN and ITS PHOTOINTERMEDIATES, K and L*. *Photochem. Photobiol.* **54**, 911–921 (1991).
4. Yi, A. *et al.* Structural Changes in an Anion Channelrhodopsin: Formation of the K and L Intermediates at 80 K. *Biochemistry* **56**, 2197–2208 (2017).
5. Gerwert, K., Freier, E. & Wolf, S. The role of protein-bound water molecules in microbial rhodopsins. *Biochim. Biophys. Acta, Bioenerg.* **1837**, 606–613 (2014).
6. Kim, Y. S. *et al.* Crystal structure of the natural anion-conducting channelrhodopsin Gt ACR1. *Nature* **561**, 343 (2018).
7. Smith, S. O. *et al.* Vibrational analysis of the all-trans-retinal chromophore in light-adapted bacteriorhodopsin. *J. Am. Chem. Soc.* **109**, 3108–3125 (1987).
8. Kuhne, J. *et al.* Unifying photocycle model for light adaptation and temporal evolution of cation conductance in channelrhodopsin-2. *Proc. Natl. Acad. Sci. U.S.A.* **116**, 9380–9389 (2019).
9. Yi, A., Mamaeva, N., Li, H., Spudich, J. L. & Rothschild, K. J. Resonance Raman Study of an Anion Channelrhodopsin: Effects of Mutations near the Retinylidene Schiff Base. *Biochemistry* **55**, 2371–2380 (2016).
10. Smith, S. O. *et al.* Vibrational analysis of the all-trans retinal protonated Schiff base. *Biophys. J.* **47**, 653–664 (1985).
11. Gerwert, K. & Siebert, F. Evidence for light-induced 13-cis, 14-s-cis isomerization in bacteriorhodopsin obtained by FTIR difference spectroscopy using isotopically labelled retinals. *EMBO J.* **5**, 805–811 (1986).
12. Govorunova, E. G., Sineshchekov, O. A., Janz, R., Liu, X. & Spudich, J. L. Natural light-gated anion channels: A family of microbial rhodopsins for advanced optogenetics. *Science* **349**, 647–650 (2015).
13. Lórenz-Fonfría, V. A. *et al.* Temporal evolution of helix hydration in a light-gated ion channel correlates with ion conductance. *Proc. Natl. Acad. Sci. U.S.A.* **112**, E5796-5804 (2015).

14. Li, H. *et al.* Crystal structure of a natural light-gated anion channelrhodopsin. *Elife* **8**, (2019).
