# Supplementary material for: Time-resolved spectroscopic and electrophysiological data reveal insights in the gating mechanism of anion channelrhodopsin
Source: Commun Biol. 2021 May 14;4:578. doi: 10.1038/s42003-021-02101-5 (PMC8121809; doi:10.1038/s42003-021-02101-5)
Supplement: Supplementary file 3 — Description of Additional Supplementary Files [file 42003_2021_2101_MOESM3_ESM.pdf]

Description of Additional Supplementary Files

File name: Supplemental Data 1

Description: The source data for the main figures is given in Supplementary Data 1.
supplementary_data_fig2_a.txt contains the source data for Figure 2a.
supplementary_data_fig2_cde.txt contains the source data for Figure 2c, 2d and 2e.
supplementary_data_fig3.txt contains the source data for Figure 3.
