# Supplementary material for: Time-resolved spectroscopic and electrophysiological data reveal insights in the gating mechanism of anion channelrhodopsin
Source: Commun Biol. 2021 May 14;4:578. doi: 10.1038/s42003-021-02101-5 (PMC8121809; doi:10.1038/s42003-021-02101-5)
Supplement: Supplementary file 5 — Reporting Summary [file 42003_2021_2101_MOESM5_ESM.pdf]

nature research

Corresponding author(s): Klaus Gerwert, Till Rudack

Last updated by author(s): Mar 23, 2021

Reporting Summary

Nature Research wishes to improve the reproducibility of the work that we publish. This form provides structure for consistency and transparency in reporting. For further information on Nature Research policies, see our Editorial Policies and the Editorial Policy Checklist.

Statistics

For all statistical analyses, confirm that the following items are present in the figure legend, table legend, main text, or Methods section.

n/a	Confirmed	
☐	☒	The exact sample size (*n*) for each experimental group/condition, given as a discrete number and unit of measurement
☐	☒	A statement on whether measurements were taken from distinct samples or whether the same sample was measured repeatedly
☒	☐	The statistical test(s) used AND whether they are one- or two-sided<br>*Only common tests should be described solely by name; describe more complex techniques in the Methods section.*
☒	☐	A description of all covariates tested
☒	☐	A description of any assumptions or corrections, such as tests of normality and adjustment for multiple comparisons
☐	☒	A full description of the statistical parameters including central tendency (e.g. means) or other basic estimates (e.g. regression coefficient) AND variation (e.g. standard deviation) or associated estimates of uncertainty (e.g. confidence intervals)
☒	☐	For null hypothesis testing, the test statistic (e.g. *F*, *t*, *r*) with confidence intervals, effect sizes, degrees of freedom and *P* value noted<br>*Give P values as exact values whenever suitable.*
☒	☐	For Bayesian analysis, information on the choice of priors and Markov chain Monte Carlo settings
☒	☐	For hierarchical and complex designs, identification of the appropriate level for tests and full reporting of outcomes
☒	☐	Estimates of effect sizes (e.g. Cohen's *d*, Pearson's *r*), indicating how they were calculated

Our web collection on statistics for biologists contains articles on many of the points above.

Software and code

Policy information about availability of computer code

Data collection	OPUS 7.2 (Bruker Corporation), Matlab R2015a (The MathWorks, Inc.), ADbasic 6 (Jäger Computergesteuerte Messtechnik GmbH), WaveRunner 6 Zi Oscilloscope Firmware version 6.6.0.5
Data analysis	IR Data analysis: OPUS 7.2 (Bruker Corporation), Matlab R2017b (The MathWorks, Inc.); Simulation data analysis: Gromacs 2019, VMD 1.9.4 PyMOL 2.2, (Schrödinger), CCP4 7.1, Maximoby (Cheops), Moby (Cheops), PyContact

For manuscripts utilizing custom algorithms or software that are central to the research but not yet described in published literature, software must be made available to editors and reviewers. We strongly encourage code deposition in a community repository (e.g. GitHub). See the Nature Research guidelines for submitting code & software for further information.

Data

Policy information about availability of data

All manuscripts must include a data availability statement. This statement should provide the following information, where applicable:

- Accession codes, unique identifiers, or web links for publicly available datasets
- A list of figures that have associated raw data
- A description of any restrictions on data availability

The raw data of the spectroscopic measurements (Figs. 2A, 3, S2, S3, S4, S5, S6, S7, S8, S9, S15, S16) as well as the simulation trajectories (Figs. 2C, 2D, 2E, S10, S11, S12, S13, S14) and input files will be provided upon individual request by the corresponding authors. The source data for the main Figures is given in Supplementary Data 1.

Field-specific reporting

Please select the one below that is the best fit for your research. If you are not sure, read the appropriate sections before making your selection.

☒ Life sciences ☐ Behavioural & social sciences ☐ Ecological, evolutionary & environmental sciences

For a reference copy of the document with all sections, see nature.com/documents/nr-reporting-summary-flat.pdf

Life sciences study design

All studies must disclose on these points even when the disclosure is negative.

Sample size	Step-scan spectra were obtained from a total of 111 measurements from 8 samples, rapid-scan spectra from about 750 measurements from 1 sample, UV/VIS spectra from about 100 measurements from 1 sample. Raman spectra were obtained by averaging 2500 individual spectra from 1 sample. The number of repetitions was adjusted according to data quality.
Data exclusions	IR-Measurements with large baseline drifts of the interferograms were excluded.
Replication	The experiments are based on purified protein samples and do not require biological replicants.
Randomization	This is not relevant here, as protein samples were analyzed and not individual subjects.
Blinding	This is not relevant here, as protein samples were analyzed and not individual subjects.

Reporting for specific materials, systems and methods

We require information from authors about some types of materials, experimental systems and methods used in many studies. Here, indicate whether each material, system or method listed is relevant to your study. If you are not sure if a list item applies to your research, read the appropriate section before selecting a response.

Materials & experimental systems

n/a	Involved in the study	
☒	☐	Antibodies
☒	☐	Eukaryotic cell lines
☒	☐	Palaeontology and archaeology
☒	☐	Animals and other organisms
☒	☐	Human research participants
☒	☐	Clinical data
☒	☐	Dual use research of concern

Methods

n/a	Involved in the study	
☒	☐	ChIP-seq
☒	☐	Flow cytometry
☒	☐	MRI-based neuroimaging

April 2020
